# Physiological and Behavioral Plasticity of the Sea Cucumber *Holothuria forskali* (Echinodermata, Holothuroidea) to Acidified Seawater

**DOI:** 10.3389/fphys.2018.01339

**Published:** 2018-09-25

**Authors:** Xiutang Yuan, Sophie J. McCoy, Yongfen Du, Stephen Widdicombe, Jason M. Hall-Spencer

**Affiliations:** ^1^Marine Biology and Ecology Research Centre, University of Plymouth, Plymouth, United Kingdom; ^2^Plymouth Marine Laboratory, Plymouth, United Kingdom; ^3^National Marine Environmental Monitoring Center, State Oceanic Administration, Dalian, China; ^4^Department of Biological Sciences, Florida State University, Tallahassee, FL, United States; ^5^School of Geography and Ocean Science, Nanjing University, Nanjing, China; ^6^Shimoda Marine Research Centre, University of Tsukuba, Tsukuba, Japan

**Keywords:** holothurians, NE Atlantic, physiological plasticity, antipredator behavior, ocean acidification

## Abstract

Research into the effects of reduced pH caused by rising CO_2_ on echinoderms has been strongly biased toward those groups which rely heavily on calcification, such as sea urchins. There is very limited information available for groups that are less reliant on calcification, such as sea cucumbers. Moreover, plasticity in physiology and behavior in holothurians, which is considered to be critical to cope with ocean acidification, remains even less understood. Here, we examined the effects of a 22-week exposure to three pH levels (pH 7.97, 7.88, and 7.79) on the responses of adult *Holothuria forskali*. This is an abundant and ecologically important sea cucumber in shallow waters of the northeast Atlantic and Mediterranean. The holothurians did not exhibit serious acidosis after a 4-week gradually decreased pH exposure, possibly due to the slow acclimation period. After an additional 18 weeks of exposure, coelomic acid–base parameters did not differ significantly among the pH treatments, whereas they were higher than in week 4. Gonad development, defense behavior, and the structure and Ca^2+^ and Mg^2+^ concentrations of calcareous endoskeleton deposited in the body wall were all unaffected by decreased levels of seawater pH. No statistical differences were found after 22 weeks, and adult *H. forskali* showed strong physiological and behavioral plasticity to the effects of lowered seawater pH. While the interpretation of our results is restricted due to small sample sizes, this first long-term study of the effects of seawater acidification on sea cucumbers revealed resilience within the wide natural range of *p*CO_2_ found in NE Atlantic coastal waters.

## Introduction

Holothurians, commonly known as sea cucumbers, are soft-bodied echinoderms that inhabit almost all marine benthic habitats, from tropical to polar areas and from intertidal zones to deep ocean trenches. Ecologically, sea cucumbers are key components of marine ecosystems. They can dominate benthic biomass and the deposit-feeders play an important role in recycling nutrients and carbonate by reworking sediments (Uthicke, 2001; Schneider et al., 2011; Vidal-Ramirez and Dove, 2016; Wolfe et al., 2018). For example, *Holothuria atra* and *Stichopus chloronotus* process an estimated 4,600 kg dry sediment (approximately the weight of the upper 5 mm of sediment in the entire area studied) annually on the Great Barrier Reef (Uthicke, 1999).

In addition to their ecological importance, holothurians are an economically important marine resource. There are approximately 1,200 known sea cucumber species, of which 70 are harvested worldwide and have been used as food for centuries throughout Asia (Yang et al., 2015), with a market value of >5 billion US$ per annum in China alone (Zhang et al., 2015). Some of them are used in integrated multi-trophic aquaculture systems worldwide as they often feed on particulate organic waste (Yuan et al., 2015b; Zamora et al., 2016).

Although the species-specific effects of ocean acidification have been widely reported for a number of heavily calcified echinoderm groups, such as sea urchins (see Dupont and Thorndyke, 2014 for review), starfish (e.g., Dupont et al., 2010a; Byrne et al., 2013; Uthicke et al., 2013) and brittle stars (e.g., Wood et al., 2008, 2010, 2011; Carey et al., 2014), their close relatives, the sea cucumbers, have been relatively understudied (Dupont et al., 2010b; Yuan et al., 2015a). To our knowledge, information on the response of sea cucumbers to lowered seawater pH is limited to studies on sperm motility in *Holothuria* spp. (Morita et al., 2010) and early development in *Apostichopus japonicus* (Yuan et al., 2015a), buffer capability of the coelomic fluid in *H. scabra* and *H. parva* (Collard et al., 2014), energetic trade-offs in physiological processes of *A. japonicus* (Yuan et al., 2016), and *trans*-generational effects of *Cucumaria frondosa* (Verkaik et al., 2016). These few studies of biological function showed that the less-calcified sea cucumbers may be more tolerant to seawater acidification than other heavily calcified echinoderms. At the community level, sea cucumbers could play an increasingly important role under near-future conditions. In the context of ocean acidification, the sea cucumber *Stichopus herrmanni* could exacerbate or buffer seawater pH by changing the ratio of total alkalinity (TA; sediment dissolution) and dissolved inorganic carbon (DIC; respiration) over diel cycles, especially in lagoons where there are low rates of seawater exchange (Schneider et al., 2011; Wolfe et al., 2018).

Many questions remain unanswered about sea cucumber responses under low pH, particularly involving their long-term physiological and behavioral plasticity. Research into the effects of ocean acidification on holothurians, as with most groups, has mainly considered short-term responses to hypercapnia, with very few addressing long-term acclimation and adaptation responses (Dupont et al., 2013). Long-term studies are important in order to avoid inaccurate assessments of the chronic effects of ocean acidification (Kurihara et al., 2013; Moulin et al., 2015; Queirós et al., 2015; Riebesell and Gattuso, 2015; Lucey et al., 2016). Organisms living in coastal or upwelling environments currently experience highly fluctuating *p*CO_2_/pH on diurnal, tidal and seasonal cycles (Hofmann et al., 2011; Wang et al., 2018). Therefore, local adaptation plays a crucial role in population sensitivity (Hofmann et al., 2014; Vargas et al., 2017), and understanding long term tolerance in these organisms is important to an assessment of their response.

The ‘Cotton Spinner’ sea cucumber, *Holothuria forskali* Delle Chiaje 1823, is a temperate species that is widely distributed on rocky bottoms and seagrass meadows in the northeast Atlantic and the Mediterranean, from the intertidal zone down to depths of about 50 m (Tuwo and Conand, 1992; Hayward and Ryland, 2017). It is a deposit feeder and acts as a key ecosystem engineer by reworking sediments (MacDonald et al., 2013). It is also valuable as seafood and has been commercially exploited in Europe in recent years, especially by aquaculture (MacDonald et al., 2013; Santos et al., 2016).

The Western English Channel is mainly <100 m deep and experiences a highly fluctuating environment (Smyth et al., 2015). Bottom waters at ‘L4’ sampling station (50° 15.00′ N, 4° 13.02′ W) often exceed 500 μatm *p*CO_2_ and peak at about 750 μatm in the autumn (**Supplementary Figure S1**); the water at the seabed (50 m depth) has a maximum, mean and minimum pH of 8.22, 8.05, and 7.73, respectively^1^. We hypothesize that epifaunal sea cucumbers that are common in shallow coastal waters are physiologically and behaviorally tolerant to decreases in pH within their natural range. In this study, we assessed the effects of long-term (22-week) exposure to decreased seawater pH on the physiology, defense behavior, and endoskeleton of *H. forskali* to investigate our hypothesis. We tested three pH levels (pH 7.97, 7.88, and 7.79, with the last two corresponding to pCO_2_ of 750 and 1,000 μatm), which capture the local natural pH variability in the Western English Channel, United Kingdom.

## Materials and Methods

### Sea Cucumber Collection and Food Preparation

*Holothuria forskali* individuals (wet body weight ∼200 g) were collected by hand using SCUBA in March and April 2015 from the Mewstone (50°18.294′ N: 004°06.310′ W) south east of Plymouth in the English Channel, from 15 to 20 m depth (water temperature 10°C, salinity 36). All individuals were kept immersed in seawater from the collection site and transferred to 1-m^3^ holding tanks at Plymouth Marine Laboratory. Here, they were held for up to 1.5 months at 10–11°C and seawater salinity 36 to acclimate to laboratory conditions (e.g., food, light, seawater pH and temperature) before the onset of our experiment. These conditions were chosen to match those at the collection site, mimicking environmental factors observed in real time at Western Channel Observatory Station L4, less than 10 km south of the collection site (Queirós et al., 2015; Smyth et al., 2015). Seawater in the recirculating tanks was obtained weekly from Station L4. Throughout the acclimation period, *H. forskali* were fed *ad labium* every day with a diet made from dried mud (65°C for 36 h) collected from Plymouth Sound and algal pellets used for marine species (New Era, United Kingdom). The coastal mud (70%) and algal pellets (30%) were well mixed with a small amount of seawater, stirred, extruded from a meat grinder into ∼3.6 mm diameter cylinders that were then dried at 65°C for 36 h. The feces and uneaten food in tanks were removed by siphoning once per week, and about 1/4 volume of seawater was exchanged at the same time.

### Seawater Acidification System

The seawater acidification system used for this study consisted of nine 1-m^3^ tanks, each filled with 700 L of seawater obtained from Station L4 and fitted with a recirculating pump and filtration system. Three effective pH_NBS_, namely, 7.97 ± 0.01, 7.88 ± 0.00, and 7.79 ± 0.00 were tested, matching current natural pH variability in the Western Channel. The three treatments were allocated at random across the nine tanks, with three replicate tanks per treatment. The seawater pH in each tank was regulated using a premixed gas system modified from Findlay et al. (2008). In brief, the three effective pH_NBS_ were achieved by pumping fresh air from outdoor (control, 7.97), or by mixing pure CO_2_ gas with CO_2_-free air using flow meters and mixing vessels, monitored with a closed path CO_2_ analyzer (Li-Cor 820, United States) and manually adjusted to maintain pCO_2_ of 750, and 1,000 μatm for pH 7.88 and 7.79 treatments, respectively. This gas was bubbled through air stones placed at the bottom of each tank. During the laboratory acclimation phase and experimental period, the tanks were maintained in a temperature controlled room with the ambient temperature set to follow the average monthly seawater temperatures observed at Station L4 (Queirós et al., 2015; Smyth et al., 2015; **Supplementary Figure S2**). Light: dark cycles were controlled by a timer that was adjusted weekly to mimic the natural seasonal day/night cycle.

Each week, 150 mL seawater from each tank was sampled and poisoned with saturated HgCl_2_ (6.9% w/w), then refrigerated in the dark prior to measurements of TA and DIC using a TA Gran Titration System (AS-ALK2, Apollo SciTech, Bogart, GA, United States) and a DIC Analyzer (AS-C3, Apollo SciTech, Bogart, GA, United States). Seawater pH was monitored three times a week using a handheld pH meter (calibrated with NBS buffers) (model 826, Metrohm, Herisau, Switzerland). The *p*CO_2_ of each tank was monitored daily using a Li-Cor instrument. Salinity and temperature were measured weekly with a salinometer (model LF197, WTW, Weilheim, Germany). Calcite and aragonite saturation states, and *p*CO_2_ were calculated based on the measured parameters (pH, salinity, temperature, and DIC) by using CO2SYS software developed by Lewis and Wallace (1998).

### Experimental Process

Four *H. forskali* were placed into each of the nine exposure tanks. A CO_2_ perturbation experiment was initiated on 25th May, 2015 and ended on 9th November, 2015. At the onset of the experimental *p*CO_2_ levels were gradually increased by around 10–20 μatm day^-1^, for a period of 4 weeks, until reaching the desired treatment levels of either 750 or 1,000 μatm. The animals were then maintained at these treatment levels for another 18 weeks.

At week 4 (the end of the gradual acidification period) and at week 22 (the end of the experiment), two individuals from each tank were sampled to measure their acid–base indices. Defense behavior was observed in week 21 whilst gonad index (GI), the Ca^2+^ and Mg^2+^ concentrations of calcareous rings and ossicles in the body wall, and the fine structure of the ossicles were determined at the end of the experiment (week 22).

### Coelomic Fluid Acid–Base Balance

*Holothuria forskali* coelomic acid–base was determined following the methods described by Spicer et al. (2011). Briefly, 100 μL of *H. forskali* coelomic fluid was extracted anaerobically on the ventral side right behind the mouth from two individuals, respectively, in each tank using a gas-tight syringe (volume = 100 μL) with a 21G gauge needle. After sampling, the sea cucumbers were gently placed back into the tanks. Total bound and dissolved CO_2_ (TCO_2_) was analyzed instantly in a subsample of coelomic fluid (vol. = 50 μL) using a TCO_2_ analyzer (956D TCO_2_ Analyzer, Corning Diagnostics, Cambridge, MA, United States), while the remaining coelomic fluid (vol. = 50 μL) was placed, within 2–3 s of sampling, in a microcentrifuge tube (volume = 1 mL, Eppendorf) and coelomic pH determined using a micro pH probe (Micro-Inlab pH combination electrode, Metter Toledo, Leicester, United Kingdom) connected to a pH meter (MP 220 pH meter, Metter Toledo, Leicester, United Kingdom).

Coelomic *p*CO_2_ and [HCO_3_^-^] in the same individual were calculated using the Henderson–Hasselbalch equation from coelomic pH and TCO_2_ determined above using Eqs. (1) and (2), respectively (Calosi et al., 2013);

(1) $$p{\text{CO}}_{2}=\text{T}{\text{CO}}_{2}/\alpha (10pH-pK_{1}^{'}+1)$$

(2) $${\text{HCO}}_{3}^{-}={\text{TCO}}_{2}-\alpha p{\text{CO}}_{2}$$

where α is the solubility coefficient of CO_2_ of sea water taken as 0.337 mmol l^-1^ kPa^-1^ at 15°C and 35 salinity (Weiss, 1974), and *pK’*_1_ is the negative log of the first apparent dissociation constant of carbonic acid taken as 6.04 at 15°C (Truchot, 1976).

### Behavioral Observations

Twenty-one weeks after the beginning of the exposures, *H. forskali* defense behavior was tested. According to Francour (1997), crabs from the genus *Cancer* attack and eat live holothurians. Therefore, we simulated a predator attack using the claw of a large *Cancer pagurus*, as this species is common in the habitat from where the *H. forskali* were collected. The predator attack mainly followed the method described by Hamel and Mercier (2000). In brief, we pinched the dorsal integument 20 times over a period of 20 s with light pressure (a test on non-experimental individuals showed that under normal pH conditions 10–15 pinches were usually enough to stimulate expulsion of the Cuvierian tubules). All tests were done between 12:00 h and 13:00 h to avoid variations due to the daily cycle of activity (Hamel and Mercier, 2000). We recorded the percentage of individuals that expelled Cuvierian tubules. This was done three times with a 3-day interval. We determined that the operation would not harm their health as we observed that the sea cucumbers often expelled tubules, and in the field they may face to predator attack as frequently as we stimulated.

### Gonad Index

The GI of the test individuals was determined according to the method of Santos et al. (2016). A longitudinal incision was made on the dorsal surface and the coelomic fluid and gonads were removed. The sex of the animals was determined using the methods of Tuwo and Conand (1992). Drained body mass (D_BM_, g) and gonad mass (G_M_, g) was measured and calculated using the following equation from Santos et al. (2016):

(3) $$\text{GI}(\%)=100*{\text{G}}_{\text{M}}/{\text{D}}_{\text{BM}}.$$

### Calcareous Rings and Ossicles

After gonad removal, the calcareous rings that surround the esophagus were dissected out individually and were placed into a 150 ml clean glass beaker. To collect the ossicles, four to five tentacles and approximately 1 cm^2^ segments from middle parts of ventral and dorsal body walls in each animal were cut and placed into beakers. 100 ml of bleach (4.6% sodium hypochlorite, Daisy, United Kingdom) were added into these beakers to digest the soft tissues for 48 h. They were then rinsed thoroughly in distilled water and dried at 65°C for 12 h. The calcareous rings or ossicles from the test animals in each tank were pooled for further analysis. Ca^2+^ and Mg^2+^ compositions of these samples was determined using an ICP Spectrometer (iCAP7400 ICP-OES, Thermo Scientific, Waltham, MA, United States). The morphology of the ossicles was assessed *via* scanning electron microscopy (Plymouth University, JSM-6610LV, JEOL Ltd., Tokyo, Japan).

### Statistical Analysis

The following statistical analyses were carried out using the SPSS17.0 software: the effect of experimental *p*CO_2_ treatment on the (1) seawater chemistry was analyzed by using repeated measure Generalized Linear Model (GLM); (2) coelomic fluid pH, *p*CO_2_ and [HCO_3_^-^], were analyzed using nested Generalized Linear Mixed Model (GLMM, with tank nested into pH treatment as an interactive random effect, R package nlme v. 3.1-131); (3) the percentage of individuals which expelled Cuvierian tubules (Arcsine-transformation was taken prior to statistical analysis) was analyzed by using repeated measures GLM; (4) GI (Arcsine-transformation was also taken) was also analyzed using nested GLMM; and (5) skeletal Ca^2+^ and Mg^2+^ concentrations were test with a one-way ANOVA. Above analyses were followed by a Tukey HSD test to determine where the differences exist. All means are presented with the standard error of mean (mean ± SEM). The level applied for significance for all statistical analyses was 5%.

## Results

### Seawater Carbonate Chemistry

There was no significant difference in seawater temperature between treatments during the experiment (*F*_1,2_= 2.200, *p* = 0.192; **Table 1** and **Supplementary Figure S2**). Seawater pH_NBS_ values in the control averaged 7.97 ± 0.01, whereas those of the two elevated *p*CO_2_ treatments were 7.88 ± 0.00 and 7.79 ± 0.00 (**Table 1** and **Supplementary Figure S3**). Elevated *p*CO_2_ exerted no significant effect on salinity (*F*_1,2_ = 0.948, *p* = 0.439) and TA (*F*_1,2_ = 0.639, *p* = 0.560), but significantly affected all other parameters of the carbonate system: pH (*F*_1,2_ = 65.395, *p* < 0.001), *p*CO_2_ (*F*_1,2_= 159.305, *p* < 0.001), Ω_ca_ (*F*_1,2_= 34.683, *p* = 0.001) and Ω_ar_ (*F*_1,2_ = 34.147, *p* = 0.001) (**Table 1**).

**Table 1:** Seawater carbonate chemistry in the seawater acidification system.

	Control	Intermediate *p*CO_2_	High *p*CO_2_
**Measured**			
Salinity	36.3 ± 0.07	36.7 ± 0.07	36.7 ± 0.08
Temperature (°C)	13.6 ± 0.08	13.9 ± 0.08	14.0 ± 0.08
pH_NBS_	7.97 ± 0.01	7.88 ± 0.00	7.79 ± 0.00
TA (μmol⋅L^-1^)	2561 ± 10	2603 ± 15	2609 ± 14
DIC (μmol⋅L^-1^)	2374 ± 7	2447 ± 10	2482 ± 15
**Calculated**			
*p*CO_2_ (μatm)	677 ± 8	914 ± 13	1133 ± 17
Ω_ca_	2.81 ± 0.04	2.37 ± 0.03	2.04 ± 0.04
Ω_ar_	1.80 ± 0.02	1.52 ± 0.02	1.31 ± 0.02

*Seawater pH (n = 162) in tanks was monitored three time a week; salinity, temperature, total alkalinity (TA), and dissolved inorganic carbon (DIC) were measured on a weekly basis (all n = 54). pCO_2_, and calcite and aragonite saturation states were calculated by using CO2SYS software (Lewis and Wallace, 1998). Values are expressed as means ± SEM.*

### Coelomic Fluid Acid–Base Balance

**FIGURE 1:**
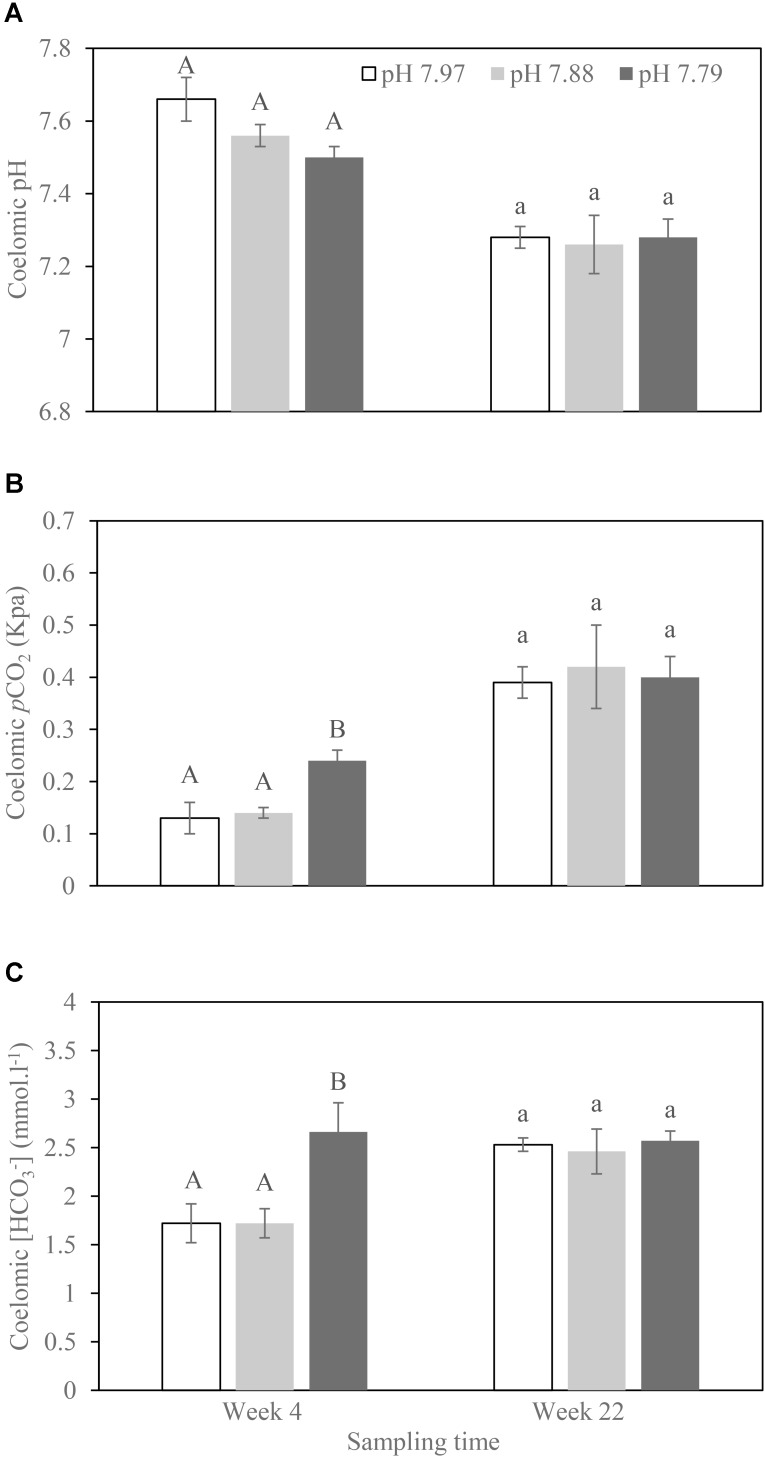
Acid–base balances (**(A)** Coelomic pH; **(B)** Coelomic pCO2; **(C)** Coelomic [HCO_3_^-^]) of the sea cucumber *H. forskali* coelomic fluid sampled at weeks 4 and 22, respectively, when exposed to pH 7.97, 7.88, and 7.79 levels. Data (expressed as mean ± SEM, *n* = 6) with different letters in the same time point identifying statistically significant differences (Tukey HSD, *p <* 0.05).

No *H. forskali* mortality occurred during the experimental period. Across all coelomic fluid acid–base parameters, we found that experimental time lowered pH_CF_ (*F*_1,27_ = 56.12; *p* < 0.001) and increased coelomic pCO_2_ and [HCO_3_^-^] (*F*_1,26_ = 68.21; *p* < 0.001; *F*_1,29_ = 8.44; *p* = 0.007; **Figure 1**). We did not detect any effect of pH treatment (*F*_1,8_ = 1.59; *p* = 0.243; *F*_1,7_ = 1.176; *p* = 0.314; *F*_1,7_ = 3.886; *p* = 0.087) nor any interactive tank effects (*p* = 1 in all models). We were unable to test for treatment differences at week 22 with a mixed effects model accounting for tank effects due to our small sample size. We note that a standard ANOVA design did not find any effect of experimental acidity treatment on coelomic fluid acid–base parameters (all *p* > 0.85), though we cannot differentiate between no effect of treatment and no detective effects due to our small sample size (**Figure 1**).

### Predator Defense Behavior

**FIGURE 2:**
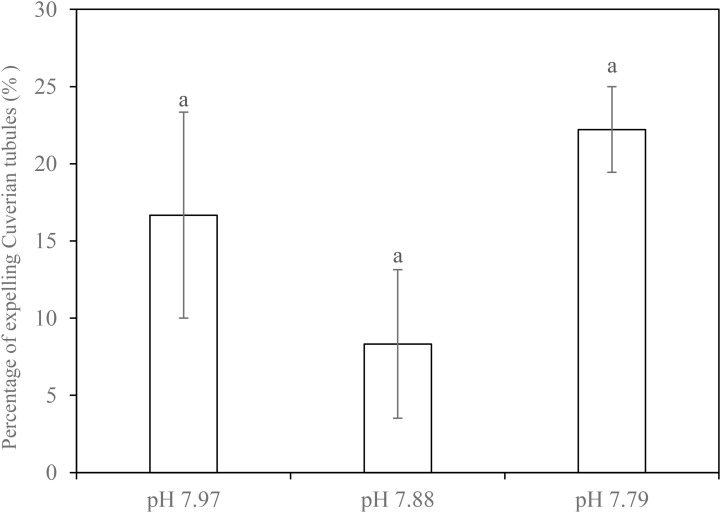
Percentage of individuals that expelled Cuvierian tubules of the sea cucumber *H. forskali* attacked using crab claw at week 21 when exposed to pH 7.97, 7.88, and 7.79 levels. Data (expressed as mean ± SEM, *n* = 3) with different letters identifies statistically significant differences (Tukey HSD, *p <* 0.05).

In simulated crab claw attacks, 8.3–22.2% of individuals released Cuvierian tubules, but there was no significant difference found in this antipredator response between the control and decreased pH treatments (*F*_1,2_ = 0.449, *p* = 0.658) (**Figure 2**).

### Gonad Index

**FIGURE 3:**
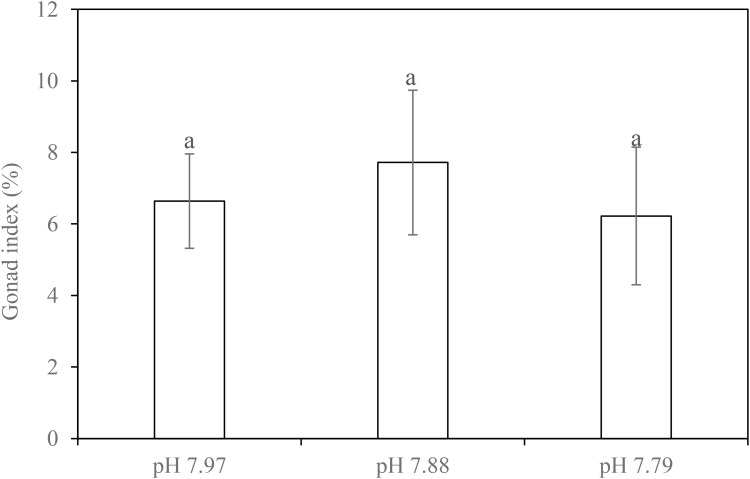
Gonad index of the sea cucumber *H. forskali* when exposed to pH 7.97, 7.88 and 7.79 levels for 22 weeks. Data (expressed as mean ± SEM, *n* = 12) with different letters identifies statistically significant differences (Tukey HSD, *p <* 0.05).

Dissections at the end of our experiment showed that 14 females, 18 males and four gender-undetermined individuals (with small gonad or without gonad that may be due to evisceration occurred during experimentation) were used in the experiment (**Supplementary Table S1**). Due to the uneven spread and undetermined gender, we did not analyze the effects of pH on GI in females and males separately. Based on nested ANOVA, there was no significant difference in GI among the treatments (*F*_2,__9_ = 0.184, *p* = 0.833) (**Figure 3**).

### Calcareous Ring and Ossicles

Based on elemental analysis, there was no significant differences in the contents of calcium (*F*_2,6_ = 0.395, *p* = 0.690 for ossicles; *F*_2,6_ = 2.496, *p* = 0.163 for calcareous rings) or magnesium (*F*_2,6_ = 0.125, *p* = 0.885 for ossicles; *F*_2,6_ = 0.812, *p* = 0.487 for calcareous rings) in ossicles and calcareous rings in *H. forskali* among treatments (**Table 2**).

**Table 2:** Concentrations of Ca^2+^ and Mg^2+^ in ossicles and calcareous rings in *H. forskali* collected at week 22 exposed to pH 7.97, 7.88, and 7.79 levels.

Effective pH	Ossicles		Calcareous rings	
	Ca^2+^	Mg^2+^	Ca^2+^	Mg^2+^
7.97	27.54 ± 0.53^a^	2.23 ± 0.04^a^	26.72 ± 1.04^a^	2.29 ± 0.10^a^
7.88	26.75 ± 0.98^a^	2.22 ± 0.05^a^	28.68 ± 0.17^a^	2.41 ± 0.04^a^
7. 79	26.61 ± 0.84^a^	2.19 ± 0.08^a^	28.14 ± 0.36^a^	2.37 ± 0.04^a^

*Values (expressed as mean ± SEM, n = 3) with different letters in the same column were significantly different from each other (p < 0.05).*

**FIGURE 4:**
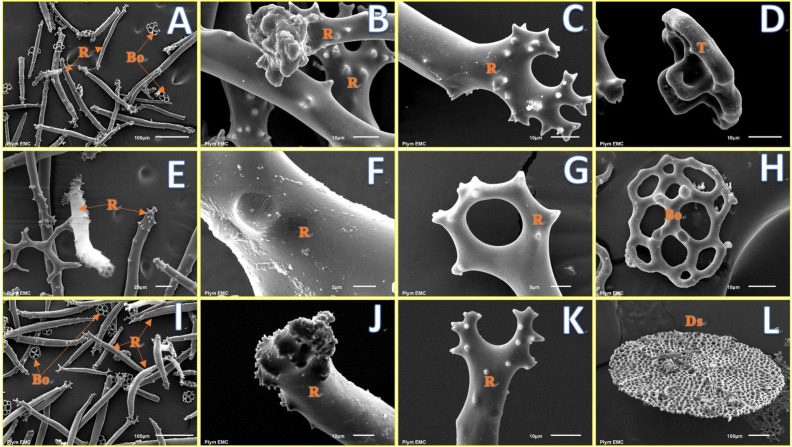
Fine structure of skeletal ossicles in the sea cucumber *H. forskali* from pH 7.97 (control, **A–D**), pH 7.88 **(E–H)**, and pH 7.79 **(I–L)** treatments after 22 weeks. **(A,E,I)** show types of ossicles from buccal tentacles; **(B,C,G,J,K)** show fine structure of the ends of rods; **(D)** shows table; **(H)** shows button; **(F)** shows fine structure of the middle part of a rod; **(L)** shows a large disk from ventral body wall. R, rod; Bo, button; T, tables; Ds, disk.

Four types of ossicles, namely, rods (**Figures 4A,E,I**), table (**Figure 4D**), button (**Figure 4H**), and disk (**Figure 4L**), were observed from the SEM images. Ossicles extracted from individuals at control (**Figures 4B–D**) and decreased pHs (**Figures 4F–H** from pH 7.88 and **Figures 4J–L** from pH 7.79) had a smooth and uniform surface, and no obvious signs of erosion in the calcareous ossicles were found at lowered pH treatments after 22 weeks of exposure.

## Discussion

The majority of laboratory studies of the effects of ocean acidification on echinoderms use short term exposures (days to weeks, without acclimation) (e.g., Miles et al., 2007; Stumpp et al., 2012; Calosi et al., 2013; Collard et al., 2014; Small et al., 2015). There are few studies on field caught sea cucumbers, due to the difficulties in keeping specimens in captivity. Although our sample sizes were small, limiting statistical power, our results provide insights into the resilience of a large shallow water species to present day variability in pH.

Adult *Holothuria forskali* did not show obvious signs of extracellular acidosis when exposed to decreased pH levels for either four or 22 weeks. There was an increase in coelomic *p*CO_2_ and [HCO_3_^-^] at week 4 in the pH 7.79 treatment, but by the end of the experiment (week 22), no readjustment in bicarbonate compensation occurred. Bicarbonate compensation may have occurred during the slow and gradual acclimation period. However, we observed significantly lower coelomic pH, and higher coelomic *p*CO_2_ and [HCO_3_^-^] in both pH treatments at week 22 than week 4. Within the scarce literature of long-term experiments on echinoderms, this phenomenon has not been found previously. In contrast, Moulin et al. (2015) observed that the coelomic pH and DIC did not differ significantly with time (measured at 3-month intervals) in the sea urchin *Echinometra mathaei* over 13 months of low-pH exposure. This may reflect different mechanisms of acid–base regulation for sea urchins and sea cucumbers, considering that the majority of sea urchins exhibit a very high buffer capacity of the coelomic fluid, while sea cucumbers have a low one and have been hypothesized to be more efficient at gas exchange (Collard et al., 2013). This low buffer capacity or high gas exchange in holothurians may mean that these coelomic parameters were more equivalent to the environment, especially at a long term conditions, for example, at our 22-week exposure to lower pH. However, a more frequent sampling with greater sample size should be undertaken before any conclusion is made for the influence of time on holothurian coelomic fluid during long term low-pH exposure. Even so, our study suggests the importance of slower acclimation to hypercapnia to avoid overestimating the effects of decreased pH in the context of ocean acidification (Moulin et al., 2015).

There is a growing realization that the indirect effects of ocean acidification will be just as important as direct effects (Gaylord et al., 2015). Trophic effects on ecological function are a poorly studied area where indirect effects of ocean acidification are likely to be important. In tropical reef systems, sediment turnover and trophic control by the sea cucumber *S. herrmanni* work to influence local carbonate chemistry (Wolfe et al., 2018), providing a potential buffering effect of acidification in tropical lagoons. As it is quite possible that trophic relationships become altered as a result of changes in predation or defense behavior under lowered pH (Gaylord et al., 2015), we simulated crab attacks in order to study the impact of lowered pH on the antipredator behavior of *H. forskali* which, like many sea cucumbers, releases Cuvierian tubules when attacked (Hamel and Mercier, 2000; Vandenspiegel et al., 2000; Castillo, 2006). The percentage of holothurians that expelled tubules was significantly unaffected by pH exposure. To our knowledge, this is the first study on the effects of decreased pH on holothurian behavior. Therefore, we suggest that this defense behavior in *H. forskali* may not be impaired by lowered pH, at least at the range of natural fluctuation in pH within its habitat. However, prey response is only one half of the story and to fully understand prey-predator interactions, further studies of the predator’s response under elevated *p*CO_2_ should also be considered.

In the NE Atlantic, adult *H. forskali* gonads mature in February and they spawn in March (Tuwo and Conand, 1992). There is a sharp fall in GI after this spawning with GI slowly recovering until the following February (Santos et al., 2016). In the present experiment, the mean GI of *H. forskali* at the end of the experiment (early November) ranged from 6.2-7.7% and this was similar to that observed in wild sea cucumbers in Portugal during November (Santos et al., 2016). While additional data on sea cucumbers is unavailable for comparison, gonad development can be negatively impacted by elevated *p*CO_2_/decreased pH levels swell beyond that examined here in some sea urchins. For example, there was a 46% reduction in GI in the sea urchin *Strongylocentrotus droebachiensis* at pH 6.98 compared to that at pH 8.10 (Siikavuopio et al., 2007). Similarly, gonad dry weight was seen to decrease by 25% and 56% in *S. droebachiensis* exposed to *p*CO_2_ treatments of 1,000 μatm and 2,800 μatm, respectively (Stumpp et al., 2012). Uthicke et al. (2014) also noted a decline in female gonads of *Echinometra* sp. held at pH 7.9 compared to those held at pH 8.1. These patterns of gonad decline, however, were not observed in the sea urchins *S. droebachiensis* and *Anthocidaris crassispina* cultured at decreased pH over a long-term period (16-month and nearly 5-month, respectively) (Dupont et al., 2013; Wang et al., 2013), pointing to variations between different urchin species and with duration of incubation.

In *H. forskali*, we did not find a significant change in GI after 22-week exposure to decreased pH, suggesting gonad development in this species was not affected by exposure to decreased seawater pH. This differs from experimental results of a north Atlantic holothurian *Cucumaria frondosa*, which was found to perform poorly in oocyte/embryo buoyancy and developmental tempo, translating into 100% mortality before the blastula stage under much lower pH conditions (pH 7.5–7.7) (Verkaik et al., 2016). Clearly, more research into the gonad development effects in holothurians is needed, considering the limited information currently available for this group.

Echinoderms have a magnesium-rich calcite skeleton, which may be vulnerable to degradation under ocean acidification conditions (Andersson et al., 2008; Dubois, 2014). In the sea cucumber *H. forskali*, however, we found no statistical differences in the concentrations of Ca^2+^ and Mg^2+^ in calcareous rings and ossicles among control and acidified groups. This is in accordance with the findings of Ca^2+^ and Mg^2+^ contents from the sea cucumber *C. frondosa* in response to lowered seawater pH (7.5–7.7) (Verkaik et al., 2016). SEM observation showed no visually obvious erosion of ossicles in individuals from lowered pH treatments. Thus, the endoskeleton of *H. forskali* has not been visibly affected by decreased pH. This can be explained in two ways: firstly, that calcite was always saturated in our tanks, unlike at CO_2_ seeps studied by Bray et al. (2014) where under saturation caused dissolution of living sea urchin spines; secondly, that urchin spines that are only covered by a thin epithelium are more prone to this threat, while holothurians are less calcified and have calcite spicules protected and embedded in their skin (Stricker, 1986). This may indicate that better physically protected skeletons are more resilient to ocean acidification.

In the present study, we emphasized the importance of conducting a long-term experiment to improve ecological relevance. Due to constraints on collecting and keeping wild specimens in aquarium holding facilities, we have a low sample size, yet our results clearly suggest resilience down to pH 7.79 for 22 weeks. We note, however, that a 22-week experiment is a short period in the lifespan of *H. forskali*, as they can live for 4–5 years. Chronic exposure to pH 7.79 throughout their life history may well prove stressful or even lethal to this species.

In summary, our study tested the hypothesis that holothurians inhabiting shallow coastal systems with highly fluctuating carbonate chemistry have high resilience to variable pH. We found that adults were resilient due to well protected calcareous skeletal deposits in their skin as well as plastic acid–base physiology and defense responses. *Holothuria forskali* currently withstand wide seasonal variability in environmental *p*CO_2_ in the Western English Channel, and this pre-adaption to large natural pH variation may explain their ability to adapt to decreased pH. Combining these results with the physiological plasticity of other sea cucumbers studied so far (Collard et al., 2014; Yuan et al., 2015a,b) highlights that these less-calcified holothurians have the potential to thrive in future, high-CO_2_ conditions. Of course, this also depends on the larval and juvenile stages of sea cucumbers being as tolerant as the adults, which is yet to be determined. In addition to expansion of studies on multiple species, populations, and life stages, data on the effects of other global stressors, such as ocean warming or benthic deoxygenation, and their impacts when acting in combination are also needed to fully appreciate holothurian responses to future ocean change.

## Ethics Statement

This study was conducted to investigate the effects of acidified seawater on physiology and behavior, including gonad development, coelomic acid-base parameters and calcareous endoskeleton fine structure of adult *H. forskali*. The gonad, coelomic fluid and calcareous endoskeleton of adults were sampled via dissection, while they were analyzed with biochemical methods as well as observation. During the experimentation and sampling, all holothurians were gently treated to minimize suffering.

## Author Contributions

XY was responsible for the original concept and was supported in the development of the concept by SW and JH-S. XY performed the experiments, strongly supported by SM and YD. XY analyzed the data and wrote the first draft. All the authors contributed to the final draft of the paper.

## Conflict of Interest Statement

The authors declare that the research was conducted in the absence of any commercial or financial relationships that could be construed as a potential conflict of interest.

## Footnotes

1 www.westernchannelobservatory.org.uk
