# Supplementary material for: Physiological and Behavioral Plasticity of the Sea Cucumber Holothuria forskali (Echinodermata, Holothuroidea) to Acidified Seawater
Source: Front Physiol. 2018 Sep 25;9:1339. doi: 10.3389/fphys.2018.01339 (PMC6167980; doi:10.3389/fphys.2018.01339)

Supplementary Material to: Frontiers in Physiology

Physiological and Behavioral Plasticity of the Sea Cucumber *Holothuria forskali* (Echinodermata, Holothuroidea) to Acidified Seawater

Xiutang Yuan[1,2,3], Sophie J. McCoy[2,4], Yongfen Du[2,6], Stephen Widdicombe[2], Jason M. Hall- Spencer[1,5]

1. Marine Biology and Ecology Research Centre, University of Plymouth, Plymouth, PL4 8AA, UK;

2. Plymouth Marine Laboratory, Plymouth, PL1 3DH, UK;

3. National Marine Environmental Monitoring Center, State Oceanic Administration, Dalian, 116023, P. R. China；

4. Department of Biological Sciences, Florida State University, Tallahassee, FL 32306-4295, USA;

5. Shimoda Marine Research Centre, University of Tsukuba, Japan；

6. School of Geography and Ocean Science, Nanjing University, Nanjing 210023, P. R. China

Table S1. Gender distribution of *H. forskali* collected off Plymouth Sound and held in nine large (700 L) laboratory tanks in pH, light and temperature controlled conditions.

Tank No.	pH treatments	Gender distribution		
		Male	Female	Undetermined
1#	7.77	2	2	0
2#	7.98	1	2	1
3#	7.88	1	2	1
4#	7.82	3	1	0
5#	7.95	3	1	0
6#	7.85	2	2	0
7#	7.79	3	1	0
8#	7.88	2	1	1
9#	8.00	1	2	1
Total		18	14	4

Fig. S1: Frequency histogram of pCO_2 (µatm) at the Western Channel Observatory site L4 for the period 2008 – 2016 (data from www.westernchannelobservatory.org.uk; Findlay pers. comm.)

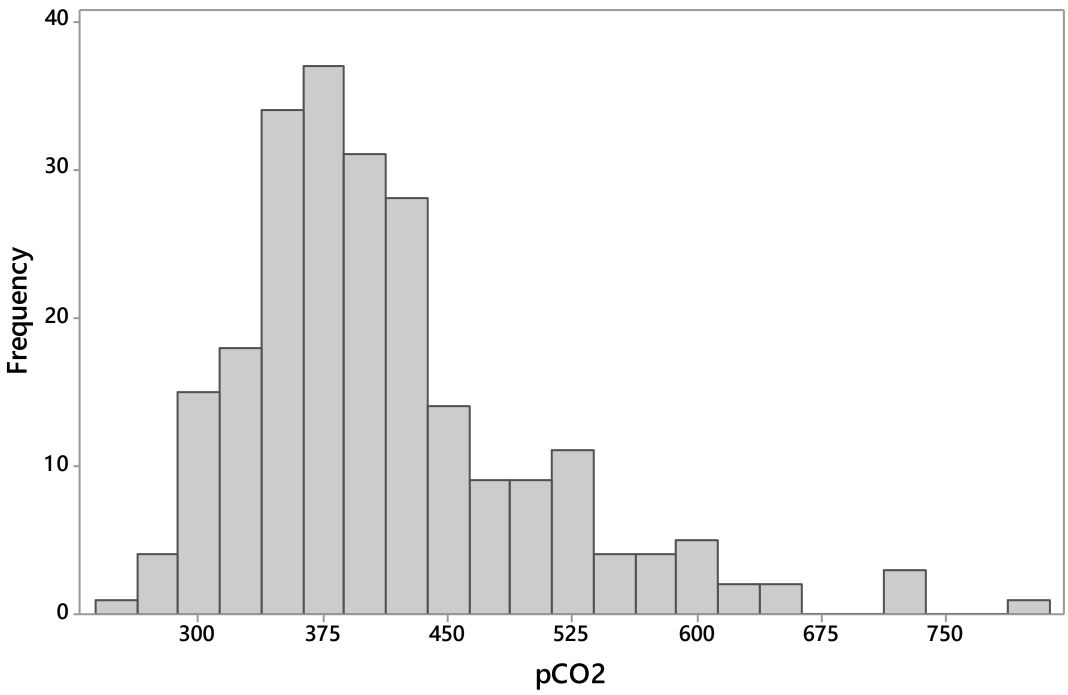

Fig. S2. Variation in seawater temperature, measured weekly, in tanks over a 22-week experimental period, matching temperature at Station L4 off Plymouth. Tanks with mean pH=7.97 shown in blue, 7.88 in orange, and 7.79 in grey. Values are expressed as means ± SEM, n=3.

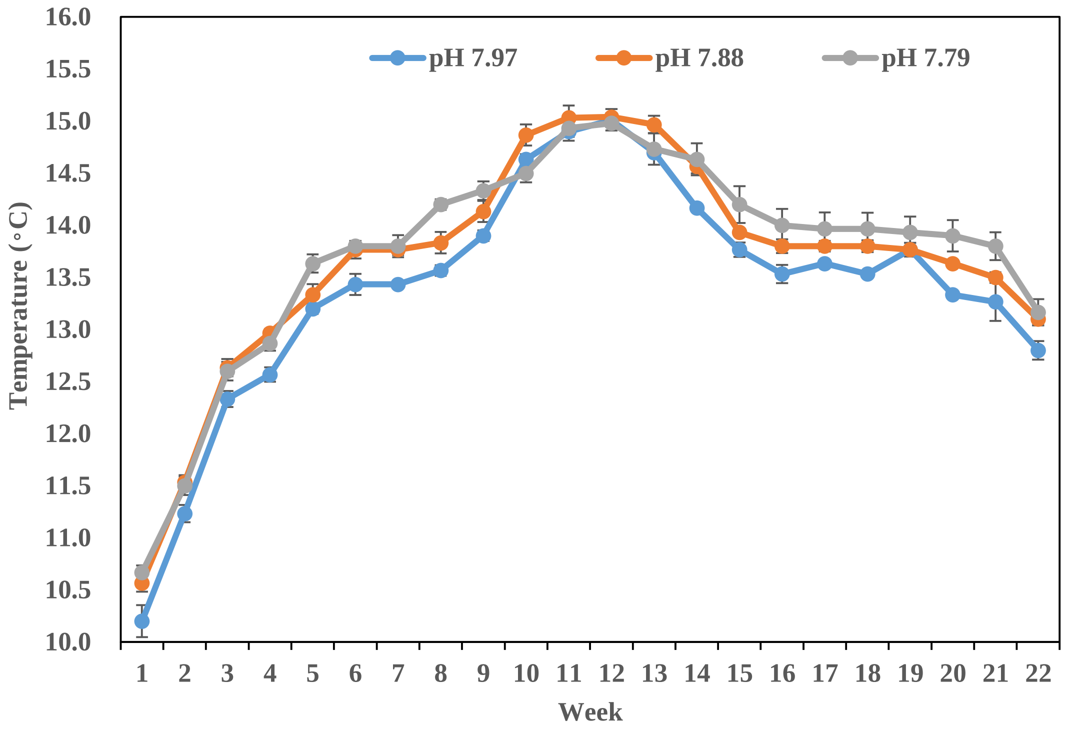

Fig. S3. Variation in seawater pH in the nine large tanks over the experimental period, starting on Day 29 after a 4-week acclimation phase. pH was measured three times a week. Tanks with mean pH=7.97 shown in blue, 7.88 in orange, and 7.79 in grey. Values are expressed as means ± SEM, n=3.

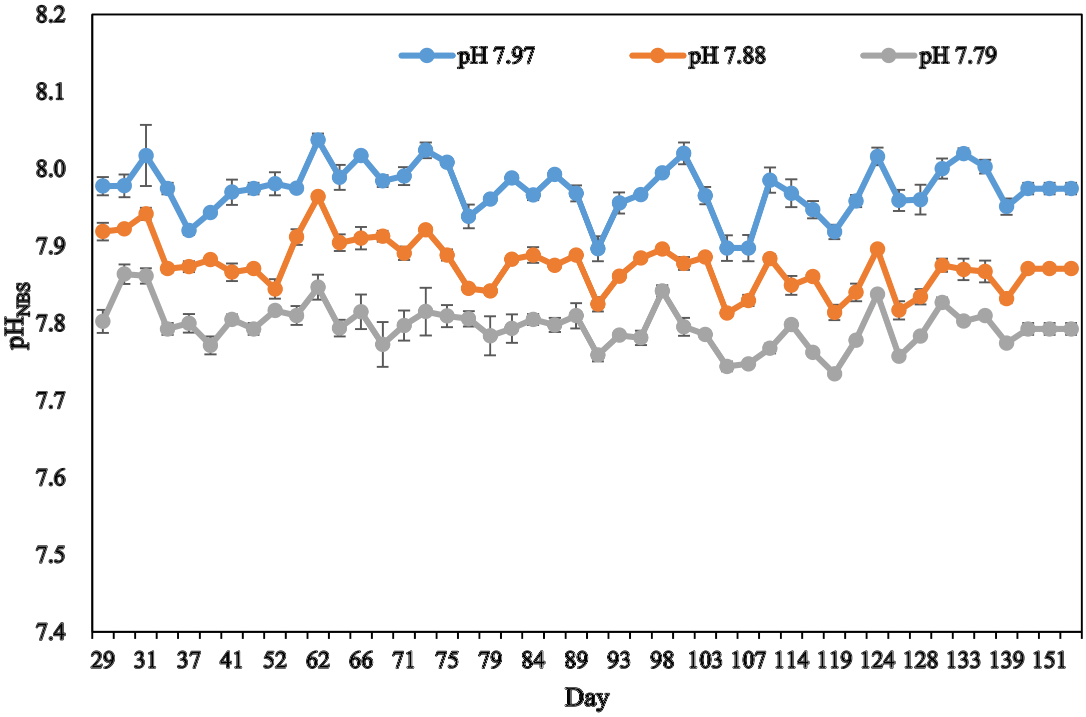

Supplement: Supplementary file 1 [file Data_Sheet_1.PDF]
